# Exploring the legacy of Ibn Imrân’s Treatise on melancholia in contemporary psychiatry

**DOI:** 10.1192/j.eurpsy.2024.1086

**Published:** 2024-08-27

**Authors:** S. Boudriga, M. Lagha, M. Methni, Y. Ben youssef, I. Ben romdhane, W. Homri, R. Labbane

**Affiliations:** Psychiatry C, Razi Hospital, Manouba, Tunisia

## Abstract

**Introduction:**

Melancholia is a concept deeply intertwined with the history of mood disorders in psychiatry. Isháq Ibn Imrân, a prominent Arab-Muslim physician of the 12th century, contributed significantly to the understanding of melancholia in his era, its. His treatise is the oldest surviving work entirely dedicated to melancholia, making it a pivotal milestone in the history of psychiatry. It is noteworthy that Ibn Imrân’s work has often been overlooked in Western psychiatry. This oversight highlights the enduring relevance of his insights within the context of modern psychiatry.

**Objectives:**

The objective of this study is to assess the clinical and therapeutic aspects delineated by Ibn Imrân in his treatise on melancholia for their contemporary accuracy and relevance within the field of modern psychiatry.

**Methods:**

The review method for the Isháq Ibn Imrân treatise involves a detailed analysis of the original Arabic text and its French translation by Adel Omrani and Radhi Jazi from the Tunisian Academy of Sciences, Letters, and Arts Beit el Hekma. This includes studying the content, structure, and historical context, as well as comparing the Arabic and French versions for accuracy.

**Results:**

The treatise is divided into two parts to clinical examination and treatment. While some of the terminology may differ from contemporary classifications, the core observations resonate with modern psychiatric knowledge. The clinical form is described as sadness, loss of pleasure, social withdrawal, dark thoughts, and loss of interest, along with somatic manifestations: sleep disturbances such as onset insomnia or hypersomnia, as well as weight loss. Additionally, perceptual disturbances, including elementary visual hallucinations (black silhouettes), are mentioned. Regarding etiologies, perinatal factors are mentioned in the treatise (“mood of the uterus”), along with six postnatal acquired causes that must be balanced in an individual: movement and rest, sleep and wakefulness, food and drink, depletion and retention, ambient air and location and psychological torment. A seasonal pattern is described, with an association between melancholia and autumn. Several clinical forms are described, with the most prominent being catatonia compared to epilepsy, in its two agitated and inhibited forms. The second part of his treatise is dedicated to treatment, focusing on individualized approaches such as talk therapy, music therapy, and dietary interventions. Ibn Imrân also describes mental strategies to correct false beliefs. For the pharmacological treatment, specific herbs has been used via oral, nasal, or intra-rectal.

**Conclusions:**

In conclusion, Isháq Ibn Imrân’s treatise on melancholia represents a timeless cornerstone in the history of psychiatry. This historical treasure serves as a reminder of the enduring quest to understand and alleviate the complexities of mental health.

**Disclosure of Interest:**

None Declared

